# Cortical signatures of wakeful somatosensory processing

**DOI:** 10.1038/s41598-018-30422-9

**Published:** 2018-08-10

**Authors:** Chenchen Song, Denise M. Piscopo, Cristopher M. Niell, Thomas Knöpfel

**Affiliations:** 1 Laboratory for Neuronal Circuit Dynamics, Imperial College London, W12 0NN London, UK; 2 Institute of Neuroscience, University of Oregon, Eugene, Oregon, 97403 USA; 3 Centre for Neurotechnology, Institute of Biomedical Engineering, Imperial College London, SW7 2AZ London, UK

## Abstract

Sensory inputs carry critical information for the survival of an organism. In mice, tactile information conveyed by the whiskers is of high behavioural relevance, and is broadcasted across cortical areas beyond the primary somatosensory cortex. Mesoscopic voltage sensitive dye imaging (VSDI) of cortical population response to whisker stimulations has shown that seemingly ‘simple’ sensory stimuli can have extended impact on cortical circuit dynamics. Here we took advantage of genetically encoded voltage indicators (GEVIs) that allow for cell type-specific monitoring of population voltage dynamics in a chronic dual-hemisphere transcranial windowed mouse preparation to directly compare the cortex-wide broadcasting of sensory information in wakening (lightly anesthetized to sedated) and awake mice. Somatosensory-evoked cortex-wide dynamics is altered across brain states, with anatomically sequential hyperpolarising activity observed in the awake cortex. GEVI imaging revealed cortical activity maps with increased specificity, high spatial coverage, and at the timescale of cortical information processing.

## Introduction

Cortical circuits integrate sensory information with memory content to guide goal-directed behaviour. While there is a consensus that this is at least one of the fundamental functions of cortical circuits, there are diverging viewpoints as to what extent components of these computational tasks are localized to specific brain areas, and how much of this computation is distributed across individual portions of the extended cortex. Early lesion studies, and anatomical and functional characterisation of input/output projections, both indicated local processing at specific brain areas, but recent research has emphasised the more distributed aspects of cortical information processing^1–5^. Indeed, a number of recent cellular resolution calcium imaging studies in awake behaving mice have identified motor information in sensory cortices and sensory information in motor cortices^6–8^.

Irrespective of the importance of local or more distributed processing, in order to trigger perception and to guide cortex-dependent behaviours, sensory information needs to be broadcasted and integrated over large portions of cortical space^9,10^. Mesoscopic imaging of genetically encoded calcium^4,5,11^ and voltage indicators^12–16^ (GECIs and GEVIs respectively) using thin-skull transcranial mouse models permits chronic cortex-wide monitoring of dynamic activity from identified cell types in awake mice. In combination with recent advances such as the mouse brain connectome and refined functional mapping, this approach provides a powerful methodological platform to achieve high spatial coverage monitoring of specific cell class populations at the timescale of synaptic signalling, allowing functional characterisation of the distribution of neuronal computation tasks for different components of behaviour. Compared to GECI imaging, GEVI-based voltage imaging has the advantages of high temporal resolution and exclusive access to hyperpolarising activity, whilst still preserving the benefits of genetically encoded optical indicators, including cell type specificity, reproducible indicator expression pattern, and the possibility of chronic preparations to allow direct within-animal longitudinal functional comparisons^12,13,17–19^.

## Results

### GEVI imaging across the dorsal view of the mouse cortex

**Figure 1:**
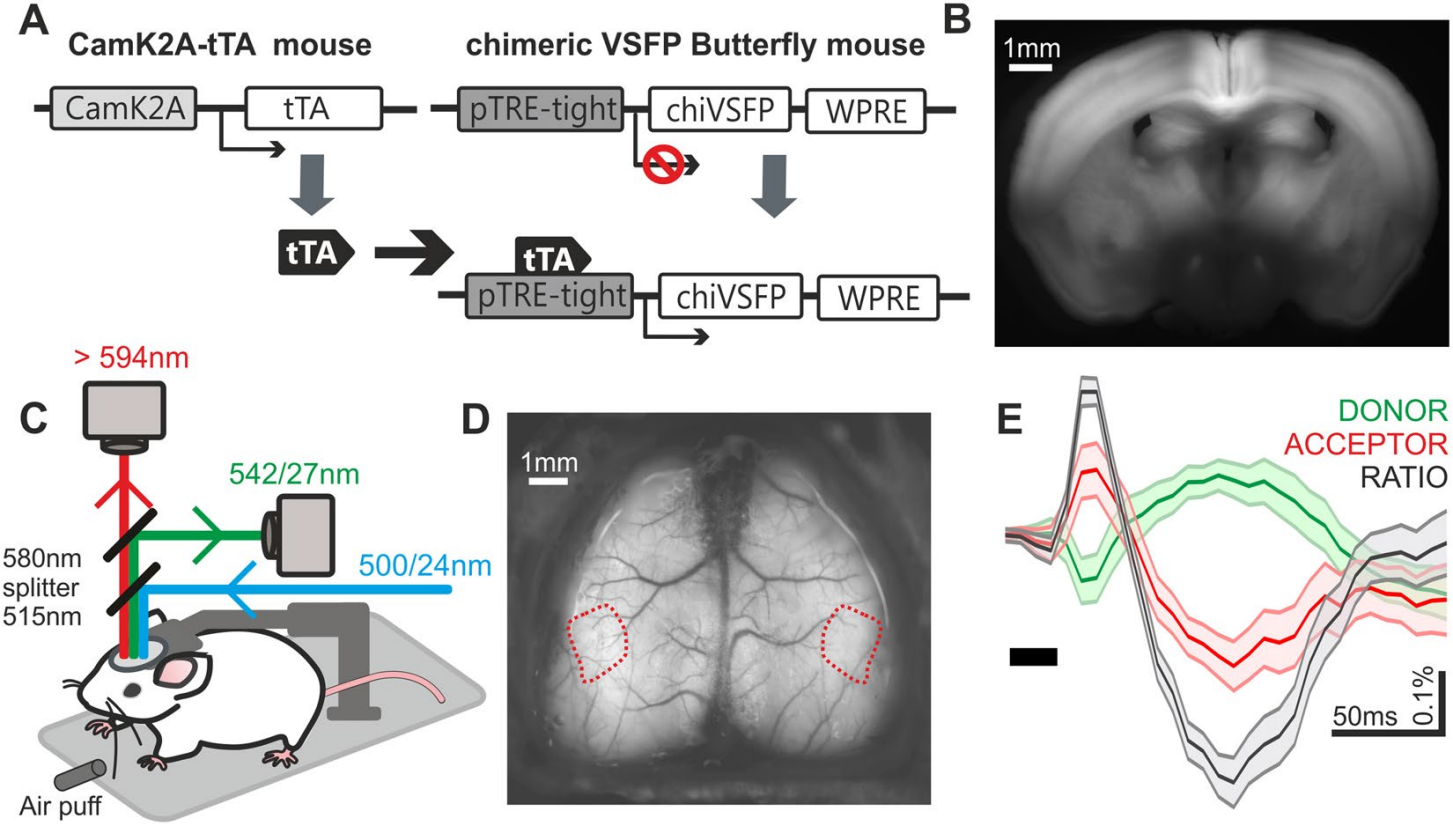
Experimental setup for GEVI-based voltage imaging. (**A**) Schematic diagram of CaMK2A promoter-controlled chimeric VSFP Butterfly (chiVSFP) expression in pyramidal neurons across all cortical layers in transgenic mice (CaMK2A-tTA;tetO-chiVSFP). (**B**) Fluorescence image of a fresh coronal brain slice from such transgenic mice. (**C**) Diagram showing head-fixation of transgenic mice implanted with a cranial window under a dual-channel widefield mesoscopic optical voltage imaging system for chronic cortex-wide monitoring of population voltage activity. Fluorescence of the differential dual emission GEVI is excited with 500 ± 12 nm light and captured at two wavelength bands (542/27 and >594 nm) by two synchronized cameras. (**D**) Dual-hemisphere thin-skull preparation with a four-arm crown for stable head-fixation. Red outlines mark primary somatosensory barrel field (SSp-bfd). (**E**) Sensory-evoked voltage response induced by an air puff directed to whiskers and recorded over the contralateral SSp-bfd as anti-correlated fluorescence intensity changes and ratiometric signal. Optical traces shown are from an example experiment at the wakening condition (50-trial average, ΔR/R for ratiometric trace with increased and decreased ΔR/R indicating depolarisation and hyperpolarisation, respectively. ΔF/F for donor and acceptor traces. Mean ± SEM).

We generated transgenic mice expressing the differential dual emission GEVI chimeric VSFP Butterfly (chiVSFP), in a transgenic configuration under the strong tetO promoter^20^. This genetic approach has been previously extensively validated to achieve strong and specific transactivator (tTA)-inducible expression. By crossing these transgenic mice with CaMK2A-tTA transgenic mice^21^, we expressed chiVSFP in excitatory pyramidal neurons across all cortical layers (CaMK2A-tTA;tetO-chiVSFP; Fig. 1A,B, Supplementary Fig. S1A). Mice were implanted with a thinned-skull cranial window for chronic (several months, Supplementary Fig. S1B) optical GEVI imaging to monitor cortex-wide pyramidal population response under head-fixation (Fig. 1C,D) at both high spatial coverage (10 × 10 mm) and temporal resolution (150 Hz frame rate). Notably, photobleaching was negligible for the illumination intensity used (0.035 mW/mm^2^), hence no photobleaching correction was needed. We used air puffs alternately to left and right whisker fields to achieve multi-whisker somatosensory stimulation. The air puffs may have evoked a small auditory response in primary auditory cortex^13^. In barbiturate anaesthetized mice, stimulus-evoked pyramidal population responses were found in primary somatosensory cortex contralateral to the site of stimulation, in the form of an early depolarisation followed by a repolarisation and subsequent rebounding depolarisation (Fig. 1D,E, Supplementary Fig. S1B). Monochromatic GEVI or GECI optical signals obtained with mesoscopic widefield imaging are known to be contaminated by intrinsic activity-dependent optical signals, and a variety of approaches have been used to account for this confound^12,20,22–25^. Here we used a differential dual emission indicator that reports membrane voltage by the anticorrelated changes in fluorescence intensity of FRET donor (mCitrine) and acceptor (mKate2) fluorescent proteins (FPs) (Fig. 1E)^12,14–16,19^. The ratiometric signal (Fig. 1E grey trace) reflects depolarising (decrease in donor and increase in acceptor FP fluorescence intensities) and hyperpolarising (increase in donor and decrease in acceptor) population membrane voltage activity.

### Processing of whisker stimulation-related information across brain states

**Figure 2:**
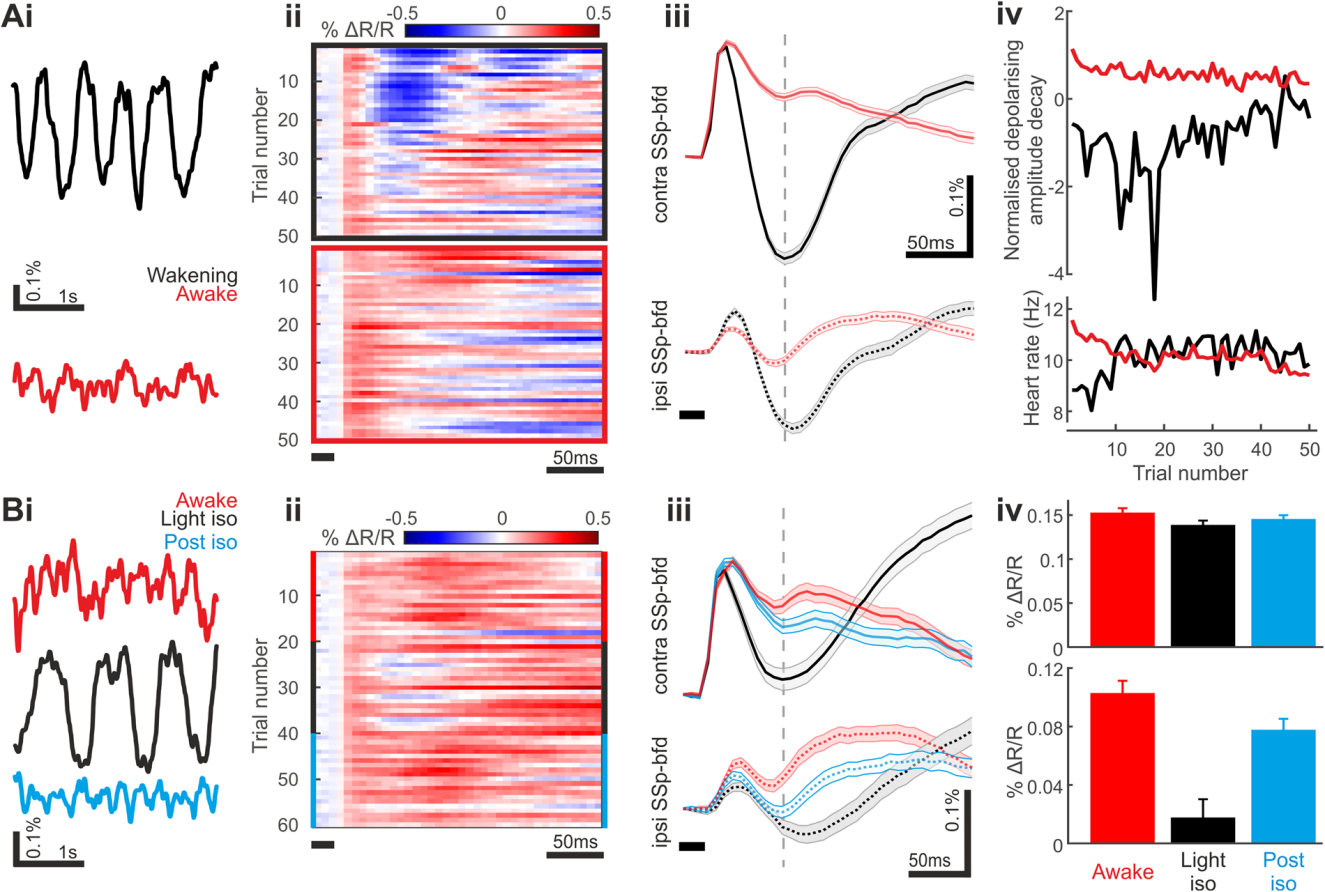
Sensory-evoked membrane voltage response of SSp-bfd pyramidal cells across brain states. (**A**) Different sensory-evoked SSp-bfd pyramidal voltage response dynamics observed during barbiturate wakening vs awake conditions. (i) Baseline optical electrocorticogram showing slow oscillating large amplitude spontaneous voltage activity during barbiturate sedation (upper, black trace), and high frequency low amplitude spontaneous fluctuations in the awake state (lower, red trace). (ii) Responses to somatosensory stimulation across trials at the wakening (upper) and awake (lower) states in contra SSp-bfd. Data are from the same animal. (iii) Somatosensory-evoked responses in contra SSp-bfd pyramidal population (upper) is triphasic during wakening (black traces) but monophasic at the awake state (red traces). Ipsi SSp-bfd (lower) displays initial depolarisation followed by repolarisation at both states. Grand average across mice and experiments shown (Wakening condition: N = 13, 5 mice, Awake condition: N = 16, 5 mice; Grand average shown). (iv) Relative decline of population membrane voltage ~50 ms after peak depolarisation (grey dashed line in A-iii) for imaging datasets in the wakening (black) and awake (red) states (upper). Changes in heartbeat frequency used as a proxy of level of sedation and alertness (lower). Grand average across mice and experiments shown. (**B**) Faster state transition using weak isoflurane sedation supports altered response dynamics across brain states. (i) Baseline optical electrocorticogram showing high frequency low amplitude spontaneous activity in the awake state (upper), slow large amplitude oscillating spontaneous activity during isoflurane sedation (middle), and the reappearance of high frequency spontaneous activity in during post-isoflurane wakefulness. (ii) Responses to somatosensory stimulation across trials during an isoflurane imaging session in contra SSp-bfd. (iii) Prolonged somatosensory-evoked responses in contra (upper) SSp-bfd at ‘awake’ and ‘post-iso’ conditions with a faster decay component in the ‘light iso’ condition. Ipsi SSp-bfd (lower) also shows greater decay component (N = 16, 5 mice; Grand average shown). (iv) Absolute somatosensory-evoked depolarising amplitude in contra SSp-bfd (upper) is similar across ‘awake’, ‘light iso’, and ‘post-iso’ conditions. Reduced repolarising amplitude in contra SSp-bfd (lower) in the ‘light iso’ condition shows faster response decay compared to both ‘awake’ and ‘post-iso’ conditions.

Using this approach, we performed GEVI imaging to monitor cortex-wide neuronal signatures of somatosensory processing at different brain states. Animals were subjected to two different experimental conditions. In the first (“wakening”) condition, mice were lightly anesthetized with a bolus injection of pentobarbiturate (eyes open, but no overt movements and limited responsiveness to touch). During the imaging session (~1.5 hr), the drug effect declined such that spontaneous motor behaviours (e.g. whisking) typically reappear towards the end of the session. To compare the responses obtained under the wakening state (ranging from light anaesthesia to approaching wakefulness with possible residual drug effects) with those in the fully awake/alert cortex, in the second condition (‘awake’), the same experimental paradigm was performed with mice well habituated to the imaging procedures. Awake data were acquired 3 or more days after the last exposure to anaesthesia/sedation. Heartbeat frequency was used as a proxy for estimating the level of sedation and wakefulness/alertness during imaging sessions. In addition to heartbeat, we monitored the global brain state of each animal via the characteristic cortex-wide voltage fluctuations (optical electrocorticogram^14^). Accordingly, we observed slow oscillatory cortical membrane voltage fluctuations during pre-stimulus baseline recordings in mice under light pentobarbital sedation (Fig. 2A-i upper), and lower amplitude fast voltage fluctuations in the fully awake cortex (Fig. 2A-i lower). Despite distinct brain states (slightly anesthetized/sedated to fully awake and alert), average sensory-evoked responses in contralateral primary somatosensory barrel cortex (contra SSp-bfd) were consistently observed with similar response latency and comparable maximum initial amplitude across brain states (wakening: 15.686 ± 0.260 ms after onset of airpuff, 0.152 ± 0.010% ΔR/R, N = 13 datasets; awake: 15.541 ± 0.171 ms, 0.159 ± 0.012% ΔR/R, N = 16 datasets; mean ± SEM; p = 0.369 for response latency, p = 0.843 for depolarising amplitude, Wilcoxon-Mann-Whitney test). However, while in the dataset obtained during wakening contra SSp-bfd displayed a triphasic response, in the awake state the same stimulus evoked a voltage transient that monophasically repolarises to baseline level (Fig. 2A-iii). This indicates that, during the first 50 ms following sensory response onset, the decay phase of the population voltage signifies somatosensory processing in the awake versus the anesthetized or wakening cortex (Fig. 2A-iii,iv). While the decay component undergoes significant change upon transition from wakening towards wakefulness (Fig. 2A-iv), the amplitude of the initial depolarising response is less affected by the change in brain state (Supplementary Fig. S2A). In the awake condition, the decay of the initial depolarising response accelerated slightly over the course of the imaging session^26,27^. Assuming that alertness of awake mice declines during the course of the imaging session (also indicated by a decline in heart rate), this indicates that a longer duration of the initial component correlates with initial increased alertness (Fig. 2A-iv).

Somatosensory inputs from the whiskers have been shown to be entirely crossed until reaching the rodent cortex^28^, hence we examined if sensory-evoked voltage responses are also represented in the ipsilateral primary somatosensory barrel cortex (ipsi SSp-bfd). We found robust ipsi SSp-bfd responses to air puffs, but more interestingly, ipsilateral responses also showed pronounced brain state-dependency (Fig. 2A-iii). In the wakening condition, ipsi SSp-bfd response components resembled those observed at the contra SSp-bfd but with an additional response delay (8.217 ± 1.806 ms difference between contra SSp-bfd and ipsi SSp-bfd response onset; mean ± SEM, p < 0.001 Wilcoxon signed rank test) and decreased response amplitude (0.059 ± 0.011% ΔR/R Fig. 2A-iii, Supplementary Fig. S1B; mean ± SEM, p < 0.001 Wilcoxon signed rank test). However, in contrast to contra SSp-bfd, as the animal transitioned from wakening towards wakefulness, we observed a decrease in the amplitude of the initial depolarising response (Supplementary Fig. S2B), accompanied by a decrease in the amplitude of the repolarising component. In the awake state, the early repolarising component is prominent in the ipsi SSp-bfd, but less pronounced in the contra SSp-bfd (Fig. 2A-iii).

Next, to both control for barbiturate-specific effects, and to control brain state at a faster timescale, we used isoflurane to modify the brain state within the imaging session - from fully awake, to light anaesthesia (‘Light iso’ condition), to subsequent recovery from isoflurane sedation (‘Post-iso awake’). We used low concentration isoflurane (0.5–0.25%), adjusted to achieve loss of active movement of facial muscles in each mouse without inducing cortical burst suppression^29,30^. The baseline optical electrocorticogram showed high frequency voltage fluctuations during both fully awake and post-isoflurane awake sessions, and high amplitude slow oscillations during light isoflurane sedation (Fig. 2B-i). Sensory-evoked responses were observed across brain states with similar response latency (Fig. 2B-ii, Awake: 15.373 ± 0.317 ms, Light iso: 15.445 ± 0.324 ms, Post-iso awake: 15.620 ± 0.121 ms; mean ± SEM, p = 0.821 ANOVA with Bonferroni’s correction). Similar to barbiturate sedation, contra SSp-bfd triphasic responses were seen during light isoflurane sedation, and comparable monophasic responses were observed during both fully awake and post-isoflurane awake states (Fig. 2B-iii). Whilst sensory-evoked population depolarising amplitude was similar across the conditions (Fig. 2B-iv, upper; Awake: 0.152 ± 0.010% ΔR/R, Light iso: 0.138 ± 0.006% ΔR/R, Post-iso awake: 0.147 ± 0.011% ΔR/R; mean ± SEM, p = 0.197 ANOVA with Bonferroni’s correction), a larger response decay (repolarisation) were observed in contra SSp-bfd during sedated state (Fig. 2B-iv, lower; Awake: 0.103 ± 0.025% ΔR/R, Light iso: 0.018 ± 0.024% ΔR/R, Post-iso awake: 0.071 ± 0.019% ΔR/R; mean ± SEM, p < 0.001 ANOVA with Bonferroni’s correction). These characteristics of cortical dynamics are consistent with those obtained when comparing awake condition with animals under barbiturate-treated condition, and hence reflect brain state and not a particular anaesthetic.

### State-dependent cortex-wide broadcasting of sensory information

**Figure 3:**
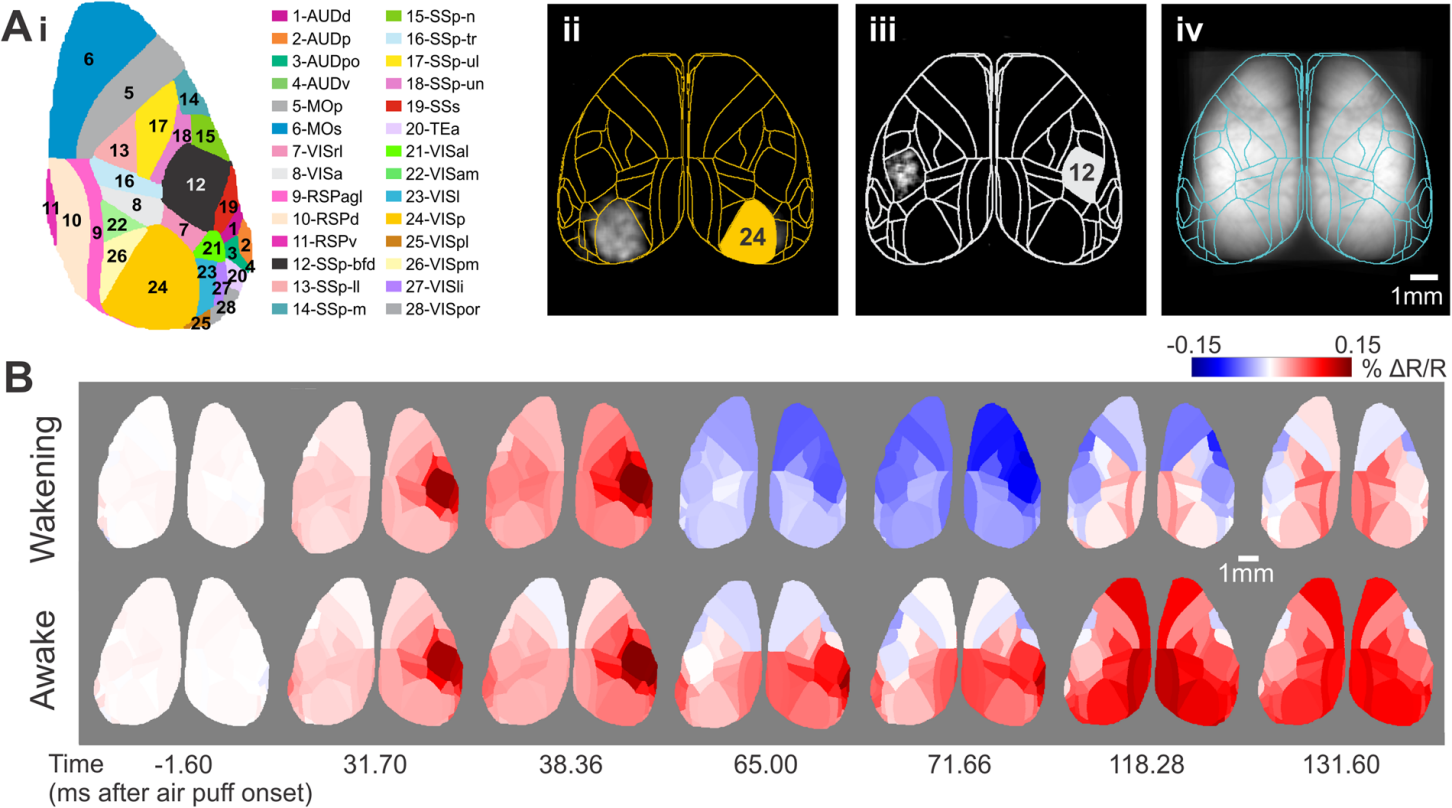
Cortex-wide pyramidal population dynamics across brain state. (**A**) (i) Brain areas within the cranial window as defined in the Allen Mouse Brain Atlas (abbreviations: 1-AUDd, Dorsal auditory area; 2-AUDp, Primary auditory area; 3-AUDpo, Posterior auditory area; 4-AUDv, Ventral auditory area; 5-MOp, Primary motor area; 6-MOs, Secondary motor area; 7-VISrl, Rostrolateral visual area; 8-VISa, Anterior area; 9-RSPagl, Retrosplenial area- lateral agranular part; 10-RSPd, Retrosplenial area- dorsal part; 11-RSPv, Retrosplenial area- ventral part; 12-SSp-bfd, Primary somatosensory area- barrel field; 13-SSp-ll, Primary somatosensory area- lower limb; 14-SSp-m, Primary somatosensory area- mouth; 15-SSp-n, Primary somatosensory area- nose; 16-SSp-tr, Primary somatosensory area- trunk; 17-SSp-ul, Primary somatosensory area- upper limb; 18-SSp-un, Primary somatosensory area- unassigned; 19-SSs, Supplemental somatosensory area; 20-TEa, Temporal association areas; 21-VISal, Anterolateral visual area; 22-VISam, Anteromedial visual area; 23-VISl, Lateral visual area; 24-VISp, Primary visual area; 25-VISpl, Posterolateral visual area; 26-VISpm, posteromedial visual area; 27-VISli, Laterointermediate area; 28-VISpor, Postrhinal area). (ii-iii) Voltage maps evoked by visual (ii) and somatosensory (iii) stimulation (normalized grey scale) registered to the Allen Mouse Brain Atlas with outline of cortex areas shown in (i). Evoked responses were thresholded at 10% of peak amplitude. Pixels below the threshold and pixels within areas outside the cortex were set to zero (represented in black). (iv) Fluorescence image of registered cranial windows averaged over 5 mice with outline of all cortical areas that project to the dorsal view. Note that lateral cortices are only partially accessed by the cranial window. (**B**) Voltage maps registered to the Allen Mouse Brain Atlas at selected times after stimulation (Wakening condition: N = 13, 5 mice; Awake condition: N = 16, 5 mice; Grand average shown. See Supplementary Fig. S4).

For sensory information to be perceived and to guide cortex-dependent behaviour, it needs to be broadcasted and integrated over larger cortical areas. To investigate this ‘broadcasting’ of neuronal information, as reflected in the activity captured from the upper cortical layers^1,31^, we first registered the two cortical hemispheres to the surface of the Allen Institute Mouse Brain Atlas (www.brain-map.org) projected to our plane of imaging (Fig. 3, Supplementary Fig. S1B; Movies S1 and S3; see Methods). We then averaged the functionally-registered population membrane voltage maps across animals in the wakening or awake experimental conditions. Spatial averages of the voltage map sequences across each cortical area were used to generate temporal sequences (“movies”) of cortical area maps colour-coded for population voltage activity (Fig. 3; Movies S2 and S4).

**Figure 4:**
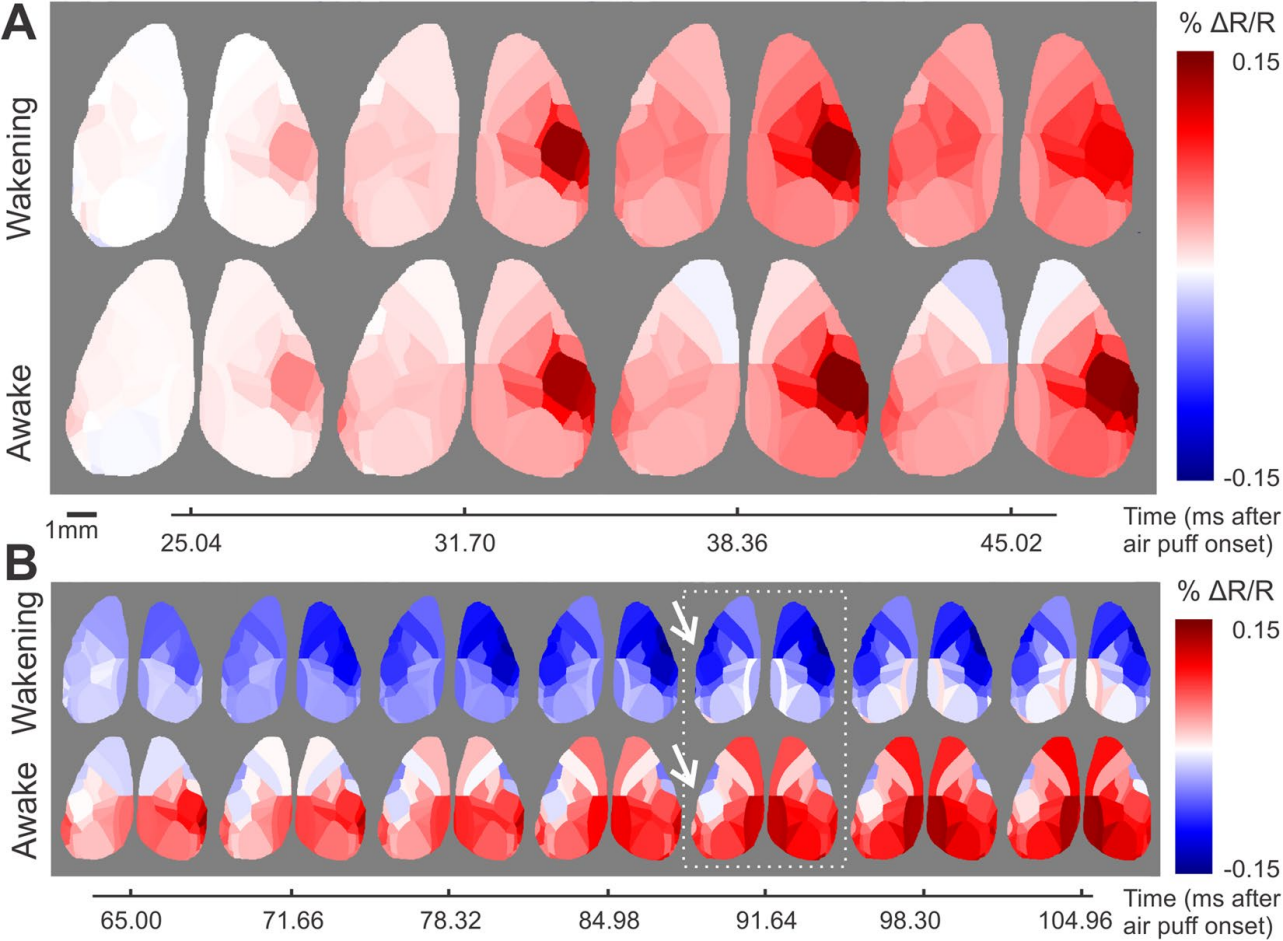
Distributed and spatially confined components of sensory processing. (**A**) Time series of voltage maps during initial depolarisation in the wakening condition (upper row; N = 13, 5 mice) and in the awake condition (lower row; N = 16, 5 mice). Same data set as used for Fig. 3B but presented at sampling frequency. (**B**) Temporal zoom into time series window of initial hyperpolarisation in wakening condition. Otherwise as in (**A**).

**Figure 5:**
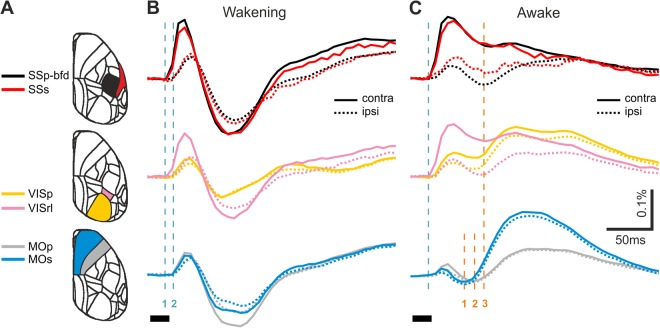
Sequential signalling in key cortical areas across brain states. (**A**) Selected brain areas, coloured corresponding to the traces shown in column (**B**,**C**). Somatosensory (upper row), visual (middle row), and motor (lower row) cortices. (**B**) Wakening condition (N = 13, 5 mice). Solid and dotted traces for contralateral and ipsilateral hemispheres respectively. Green dashed lines indicate onset of initial depolarisation measured over SSp-bfd (green - 1), followed by delayed depolarising response in other cortical regions (green - 2). For clarity, only the grand average trace is shown. (**C**) Awake condition (N = 16, 5 mice). Green dashed line indicates onset of initial depolarisation measured over SSp-bfd. Orange dashed lines highlight the sequential timing of maximal hyperpolarisation in secondary motor (MOs; orange - 1), primary motor (MOp; orange - 2) and sensory cortices (orange - 3).

These high spatial coverage movies allowed us to visualise and analyse the electrical activity propagation over both cortical hemispheres following sensory stimulation. Notably, as we averaged across animals and across left and (mirrored, see Methods) right stimulation our analysis focuses on typical (average) response patterns. This approach is reasonable as traces from individual mice and stimulation sides vary around their average (Supplementary Fig. S3). Both in the wakening and the awake condition, we observed the initial depolarising activity rapidly evolving in the contra SSp-bfd pyramidal population from where it spreads over the entire sensory cortices (Fig. 3B; Movies S1–S4). Importantly, the initial depolarisation seen at the side ipsilateral to air puff stimulation is not confined to the barrel field (ipsi SSp-bfd) but is observed with comparable amplitudes across the entire somatosensory cortices of both hemispheres. The globalisation of the initial “ipsi SSp-bfd” signal occurred regardless of the brain state, both in the wakening (Fig. 4A upper) and in the awake state (Fig. 4A lower). The hyperpolarising response following the initial depolarisation – which for contra SSp-bfd and secondary somatosensory cortex (SSs; see below) is observed only during wakening — is similarly seen over the entire sensory cortices. However, in the ipsilateral hemisphere the hyperpolarising signal component appears to dominate in ipsilateral facial somatosensory areas (SSp-bfd, SSp-m and SSp-n) in both wakening and awake states (Figs 4B, 5). In the wakening condition, this hyperpolarising response distributes across cortical space, but in the awake state, we observe sequential hyperpolarising activity across more restricted cortical regions (Fig. 5C, orange dashed lines), with motor cortices achieving comparable hyperpolarising amplitudes to ipsi SSp-bfd but with an earlier onset (Fig. 5C; Movies S3, S5). As the animal transitioned from wakening towards wakefulness, similar to SSp-bfd (Fig. 2, Supplementary Fig. S2), we observed a decrease in the hyperpolarising amplitudes in sensory and motor cortices across both cortical hemispheres (Supplementary Fig. S5B).

In the awake condition, a prominent largely bilaterally symmetrical hyperpolarising response emerged first in the secondary motor cortices (MOs), and thereafter sequentially in the primary motor cortices (MOp) and mouth and nose primary somatosensory areas (SSp-m and SSp-n respectively; Movies S3 and S4). In the ipsilateral hemisphere, this hyperpolarising response was then extended to ipsi SSp-bfd (Figs. 3B and 5; Movies S3 and S4). Also similar to SSp-bfd (Fig. 2, Supplementary Figs S2 and S5), hyperpolarising amplitude gradually increased in both sensory and motor cortices over the course of the imaging session (Supplementary Fig. S5).

Sensory-evoked response time course in SSs largely resembles those of SSp-bfd for both cortical hemispheres (Fig. 5), with similar initial depolarising onset, peak time and response amplitude in both states. Also similar to SSp-bfd, we observed relatively constant initial depolarising amplitude in contralateral SSs through the transition from wakening towards wakefulness (Supplementary Fig. S5B), and accelerated decay over the imaging session in the fully awake state (Supplementary Fig. S5C). Interestingly, in the contralateral hemisphere, a similar response time course is also observed in the rostrolateral visual area (VISrl; Fig. 5, Supplementary Fig. S5). Recent GECI imaging experiments have refined and extended retinotopic maps in the mouse cortex that also intrude into the somatosensory cortex^32^. Along with our current observations this indicates a previously underestimated allocation of cortical resources for multisensory integration, and suggests a reciprocal visual and somatosensory processing in VISrl and primary somatosensory cortex.

In contrast to contralateral SSp-bfd, SSs and VISrl, during the same transition sequence (light anaesthesia to wakening), we observed a decrease in the initial depolarising amplitude in both primary visual (VISp) and motor cortices (MOp and MOs, Supplementary Fig. S5B). This decrease was also observed over the duration of the imaging session for the awake condition (Supplementary Fig. S5C). As the mice are likely in a more increased state of alertness at the beginning than at the end of the imaging session (see the gradual ramping down in heartbeat frequency; Fig. 2B-iv red trace; 6 A upper), together, these observations indicate sensory-evoked cortex-wide activity broadcasting is largely dependent on fine gradations of brain state, including alertness.

### Secondary depolarising response in the awake state

**Figure 6:**
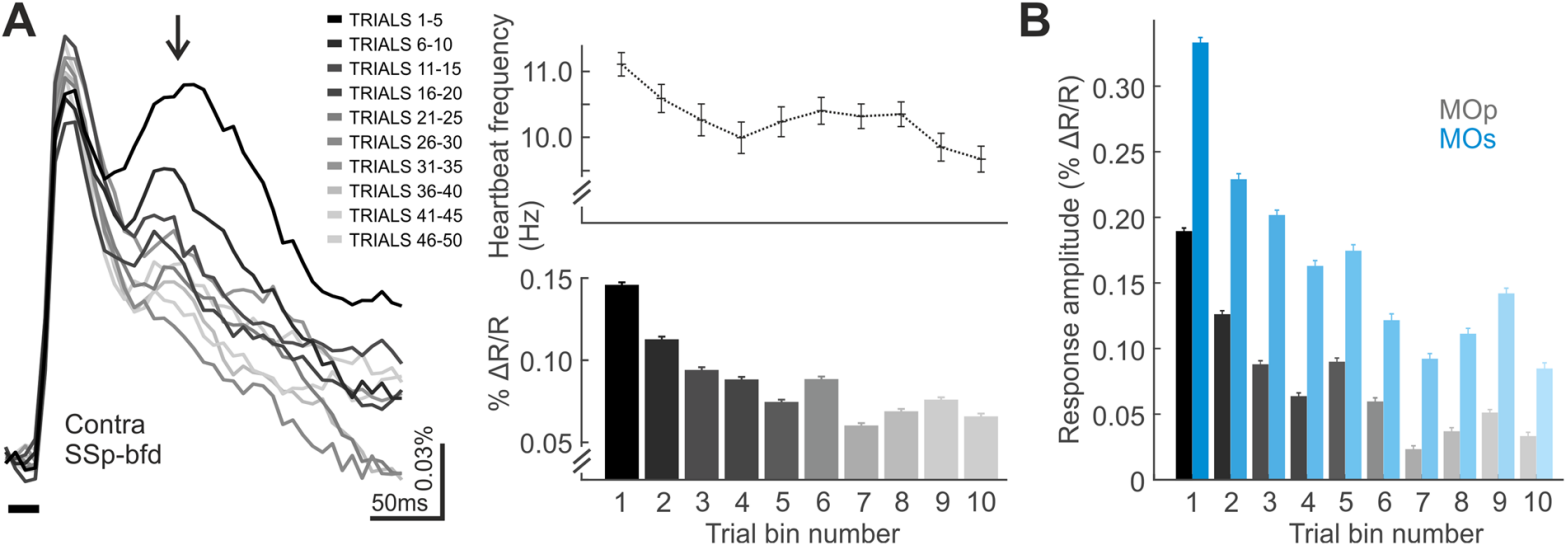
Secondary depolarising component in the awake state. (**A**) Left: Secondary depolarising response component recorded over contra SSp-bfd ~50–80 ms (arrow) following the initial depolarisation (5-trial bins grand average; for clarity, only trace average is shown). (Right: upper) Average heartbeat frequency per trial bin (dotted trace, N = 16 datasets, mean ± SEM). (lower) Absolute amplitude of the secondary depolarising peak per trial bin (N = 16 datasets, mean ± SEM). (**B**) Absolute secondary depolarising component in contra MOp and MOs (5-trial grand average, N = 16 datasets, mean ± SEM) and relative secondary response.

Secondary depolarising responses in the sensory cortices have been associated with perceptual processing of sensory information^33^. Despite the passive nature of our air puff stimulus, we observed a secondary depolarising response ~80 ms after the initial depolarising response in the awake contra SSp-bfd that is particularly prominent in the earlier trials of the awake condition imaging sessions (Fig. 6A). The reduction in the amplitude of this secondary depolarising component through the course of the imaging session likely reflects a decreased level of alertness or arousal, as also reflected in the decrease in heart rate^26,27^ (Fig. 2A-iv, 6A upper). Interestingly, a prominent secondary depolarising component is also observed in the motor cortices (MOp and MOs, Fig. 6B), where the initial response decreases in absolute amplitude through the imaging session in a similar fashion. This decrease in secondary depolarisation over the course of the imaging session in both somatosensory and motor cortices suggests that the recorded signals relate to higher level cortex-wide sensorimotor integration (including changes in behavioural adaptation) occurring at elevated levels of alertness^34–36^.

## Discussion

Distributed information processing in the cortex calls for large-scale mesoscopic monitoring of dynamic neuronal activity at the timescale of behaviour. Here we used a novel transgenic mouse line expressing the genetically encoded voltage indicator (GEVI) chimeric VSFP Butterfly (chiVSFP) under the tightly regulated tetO (TRE) promoter, and achieved chiVSFP expression in pyramidal neurons for cell class-specific monitoring of cortical electrical activity in a chronic preparation. We first show that multi-whisker somatosensory stimulation triggers different sensory processing dynamics in the primary somatosensory cortices between the wakening and fully awake/alert brain states. We then analysed how primary sensory information is broadcasted from the primary somatosensory cortex over both cortical hemispheres in both brain state conditions. Dual-hemisphere monitoring showed a novel cortex-wide hyperpolarising activity following the initial sensory-evoked depolarising response in the wakening state. In contrast, in the awake state, sensory-evoked activity is more spatially discrete, with a prominent bilateral hyperpolarising response that emerges first over the motor cortices and subsequently in the sensory cortices in an anatomically sequential manner. Additionally, we observed secondary response components that may be associated with higher order sensory processing.

To guide optimal behaviours, the integration of sensory information with other cognitive components such as memory, attention, and motivation is crucial. Accruing evidence currently emphasises the recruitment of spatially distant cortical regions to achieve goal-directed behaviour^3–5,10^, but even the processing and integration of sensory information alone recruits dynamic activity of different regions across a large cortical space^37^. Furthermore, functional and dynamic neuronal computations also involve hyperpolarising activity, as well as subthreshold voltage fluctuations, both of which cannot be captured using the mainstream GECI-based imaging approaches. To fully understand the circuit mechanisms underlying behaviour it is necessary to directly monitor neuronal activity with high temporal resolution and high spatial coverage. Voltage sensitive dye imaging (VSDI) has advanced considerably towards achieving this goal^31,38^, but is methodologically blind to cellular identity and unsuitable for studies extending over several weeks. Recent progress in GEVI development and GEVI-based mesoscopic voltage imaging approach provides an exclusive opportunity for observing cell type-specific population electrical activity with sufficient spatiotemporal resolution and coverage^12–15,17,18,27,39,40^. We note that, single-photon imaging is likely dominated by activity in the superficial layers due to penetrance of visible light in tissue.

The new transgenic mouse line (tetO-chiVSFP) expresses the ratiometric FRET-based GEVI chiVSFP under the control of a transactivator (tTA). CaMK2A-tTA;tetO-chiVSFP mice expressed chiVSFP in pyramidal neurons (under the CaMK2A promoter) across cortical layers (Fig. 1, Supplementary Fig. S1A). The mouse model showed structural and functional stability for up to 13 months following thin-skull cranial window implant (Supplementary Fig. S1B), demonstrating that GEVIs allow longitudinal monitoring of neuronal activity *in vivo*. Cortex-wide monitoring and the registration of cortical regions onto connectivity maps, such as the Allen Institute Mouse Brain Atlas used here, allows standardised fluorescence signal attribution across animals. Optical monitoring using differential dual emission GEVIs (Fig. 1E) offers the advantage of improved separation of haemodynamic and voltage signals, which can be problematic for monochromatic *in vivo* widefield optical imaging experiments in the mammalian brain^12,20,22–25^.

In the wakening condition, GEVI imaging of sensory-evoked population voltage responses from pyramidal neurons revealed triphasic responses in contra SSp-bfd, similar to previous indications from VSDI^37,41–43^, but with the additional circuit delineation of pyramidal cell types (Fig. 2). In the awake state, on the other hand, the sensory-evoked depolarising response in contra SSp-bfd monophasically returns to baseline (Fig. 2A), and this extended response duration in the awake cortex also is in line with observations from VSDI single whisker stimulation experiments^44^. Using isoflurane, we confirmed this altered population voltage response is not anaesthetic-specific (Fig. 2B).

Extending previous work, we found whisker stimulation-evoked responses with early onset similar to that of contra SSp-bfd in two adjacent cortical areas, SSs and VISrl. SSs displays a sensory-evoked response similar to that of SSp-bfd under both wakening and awake conditions (Figs 3 and 5). In line with existing cellular electrophysiology observations^45^, the similar response onset delay indicates parallel thalamic inputs into SSs. Previous experiments inactivating the primary somatosensory area have shown to modulate SSs response properties. Since our experimental design does not involve decision making or behavioural output, the brain state dependent signalling in SSp-bfd and SSs represents sensory processing.

The early onset response in VISrl is distinctly different from the activity in VISp (Fig. 5), indicating that VISrl response reflects, at least in part, primary somatosensory processing rather than multimodal sensory integration or secondary processing of visual information. Existing evidence from cellular-resolution electrophysiology and calcium dye imaging has observed multisensory circuitries in VISrl^46^, and recent GECI functional imaging experiments have also identified additional cortical areas in the retinotopic map that intrude into other regions including the somatosensory cortex^32^. To be cautious, we like to note that we cannot exclude scattered responses from barrel cortex or imperfect mapping of cortical space to the Allen Mouse Brain Atlas. Together with our current observations, this highlights the distributed nature of sensory information processing involving multiple cortical areas.

The high spatial coverage of mesoscopic imaging allows us to monitor dynamic activity changes simultaneously across both hemispheres (Figs 3–5; Movies S1–S4). As somatosensory inputs in rodents are entirely crossed until reaching the cortex, we anticipated an ipsilateral homotopic echo response, as cellular-level electrophysiology observations indicated^28^. In contrast, in our study, the initial depolarising response in the ipsilateral hemisphere is not restricted to SSp-bfd in both wakening and awake conditions (Figs 3 and 4). Under wakening condition, the initial cortex-wide depolarising response reaches similar amplitudes across sensory and motor cortices in the ipsilateral hemisphere (Figs 3–5; Movies S1 and S2). This cortex-wide broadcasting could be facilitated by slow wave activity present during the sedated brain state^47^, and this could be restricted by the enhanced synaptic inhibition at wakefulness^48^.

In the awake state, the sensory-evoked response pattern is spatially and temporally more segregated in line with the EEG-derived concept of desynchronization. In contrast to the cortex-wide hyperpolarising period in the wakening condition, in the awake state we observed a chain of sequential hyperpolarising activities engaging different anatomically defined regions, from MOs, MOp, to primary sensory regions (SSp-n and SSp-m), with the involvement of SSp-bfd only in the ipsilateral hemisphere (Figs 3–5, Movies S3 and S4). Despite the hemispheric differences in the sensory cortices, largely homotopic responses are observed in the motor cortices across brain states (Figs 3–6; Movies S1–S4). Motor cortices also display a response that is dynamically distinct from those of the sensory cortices, especially in the awake state (Figs 4–5). It shall again be noted that in contrast to other studies using goal-directed paradigms, no goal-related motor actions are required in the current study, therefore, although we do not exclude spontaneous movements, our observations are not confounded by activities related to goal-related decisions or generation of decision-related motor commands. We do not exclude motor reactions, and such motor response may require perception.

Classical electrophysiology and imaging experiments have established that different brain states such as active, resting, sedation or sleep are reflected by characteristic features of neuronal circuit dynamics^1,35,49–55^. Using cell class-specific GEVI imaging we extended these studies showing how somatosensory-evoked response dynamics of pyramidal neurons reflects the level of sedation, wakefulness and arousal. Using heartbeat frequency as a proxy of the level of sedation and arousal^26,27^ (Figs 2A-iv, 6A), we found that, through the transition from light anaesthesia to awake, reduced hyperpolarisation across multiple sensory and motor cortices is accompanied with decreased initial peak amplitude particularly in ipsilateral sensory cortices, and bilateral motor cortices (Supplementary Fig. S5). Interestingly, as the arousal/alertness decreases through the course of the awake imaging session, we observed a decrease in the initial peak amplitude in multiple regions amongst sensory and motor cortices (Supplementary Fig. S5), while the initial peak amplitude in SSp-bfd (Fig. 2, Supplementary Fig. S2) and SSs remained largely constant (and similar to that observed under wakening). This indicates increased cortical broadcasting during increased alertness. These changes in cortex-wide broadcasting likely requires fine-tuned state dependent inhibitory activity^48,56–60^. In line with this idea, we observed a gradual increase in sensory-evoked hyperpolarising amplitude in multiple sensory and motor regions across both hemispheres through the awake imaging session (Supplementary Fig. S5). Furthermore, such fine-tuned inhibition would also contribute to the sensory-evoked sequential pyramidal population hyperpolarising activity from motor to somatosensory cortices in the awake cortex (Figs 3–5).

Cellular-level observations combined with goal-directed behavioural paradigms had indicated a secondary depolarising response component associated with the conscious perception of sensory input^33^. However, since this earlier study included a motor task to implement readout for the perception, the secondary response may be related to the decision-making process or motor task itself. Here we observed a prominent secondary depolarising component in both the somatosensory (Fig. 6A) and motor cortices (Fig. 6B) that likely reflects alertness-dependent processing with sensorimotor integration (Fig. 6).

Our study highlights the potential of brain-wide voltage imaging approaches and sets a foundation for a number of future studies, among which most pressing is the role of different classes of GABAergic cells in sensorimotor integration^59,61,62^.

## Materials and Methods

### Animals

Generation of transgenic mice (tetO-chiVSFP) expressing the GEVI chimeric VSFP Butterfly under the control of a tetracycline response element (TRE) was performed in the University of Oregon, USA. Animals used for transgenic mice generation were maintained in the animal facility at the University of Oregon, and used in accordance with protocols approved by the University of Oregon Institutional Animal Care and Use Committee.

tetO-chiVSFP mice were generated by injection of a gel-purified linear DNA fragment into fertilized oocytes. Embryos for injection were obtained by mating (C57BL/6J and DBA) F1 hybrids. 5 transgenic founders were crossed with mice expressing tTA under the control of the alpha isoform of calcium/calmodulin-dependent protein kinase type II alpha chain (CaMK2A) promoter on the C57BL/6J background (stock number 007004; The Jackson Laboratory, Bar Harbor, ME^21^). Following confirmation of transgene incorporation by PCR and Mendelian ratios of germline transmission, transgene expression was screened. In one founder line, we found strong expression throughout cortex as seen by fluorescence in histological sections and by mRNA situ hybridization, with a distribution that approximates the expression of the tTA driver. No fluorescence was observed in mice that were not crossed to the tTA driver line, confirming that expression is dependent on the driver. Expression was found across the cortex, selectively in pyramidal neurons in all cortical layers.

CaMK2A-tTA;tetO-chiVSFP mice used for imaging experiments (aged 3–13 months, either sex) were maintained at Imperial College London, UK. All experimental procedures performed at Imperial College London were in accordance with the UK Animal Scientific Procedures Act (1986) under Home Office Personal and Project licences following appropriate ethical review.

### Surgery

All mice underwent surgical anaesthesia for cranial window implantation surgery as described previously^14,15^ with minor modifications. In brief, a metal head-plate (Fig. 1C) was implanted onto intact thinned mouse skull and secured using a self-cure adhesive resin cement (Super-Bond C&B, Sun Medical, Japan). The thinned skull was reinforced by a cover of resin cement topped by a thin layer of clear nail polish. All mice were allowed to recover for at least 7 days after surgery prior to voltage imaging sessions and behavioural habituation.

### Optical voltage imaging

Image acquisition was performed with a dual emission widefield epifluorescence macroscope equipped with two synchronised CMOS cameras in global shutter mode (Basler AG), using high power halogen lamps (Moritex, BrainVision) and the following optics (Semrock): mCitrine (donor) excitation 500/24, mCitrine emission FF01-542/27, mKate2 emission BLP01-594R-25, excitation beam splitter 515LP, and detection beam splitter 580LP.

Image sequences of 10-s duration were acquired at a frame rate of 150 Hz, at 400 × 304 pixel 12 bit resolutions, 6 ms exposure time. It may be noted that CMOS cameras integrate over exposure time, thus signals faster than 150 Hz will not escape detection. Imaging with fast low molecular weight dyes in acute preparations has shown that imaging at 150 Hz provides sufficient temporal resolution for optical recordings of the population membrane voltage transients studies here^1,31,44^. 50 trials per dataset (N) were acquired for wakening or awake conditions, and 60 trials for dataset (N) for isofluorane (20 trials each for awake/light iso/post-iso conditions) experiments.

### Animal sedation and habituation

For imaging sessions in the wakening state, 30 mg/kg pentobarbital sodium (Sigma) in 0.9% saline (i.p. bolus injection) was administered <5 min prior to the start of the imaging session. For the light isoflurane anaesthetised condition, 0.5–0.25% isoflurane in oxygen was used.

Animals were handled prior to behavioural habituation. For habituation sessions, animals recovered from decreasing levels of light sedation under head fixation for up to three sessions without sensory stimulation. Animals were then head-fixed awake for increasing durations for up to four additional habituation sessions without sensory stimulation.

### Whisker stimulation

Multiple whiskers were deflected simultaneously using a brief air puff delivered from a picospritzer (Picospritzer II, Parker Hannifin), via 2 mm diameter plastic tubes placed at approximately 2 cm in front of the whiskers on both sides, and delivered similar strength air puffs (10 psi, 20 msec duration). Air puffs were given in alternating conditions between left- and right-stimulation, with ~30 sec inter-trial-interval, maximum 60 trials per side per day. Whiskers were trimmed to approximately 5 mm length prior to each experiment.

### Histology and anatomy

Animals were terminally anaesthetised with ketamine/xylazine and transcardially perfused with phosphate buffered saline (PBS, Sigma) followed by 4% paraformaldehyde (Sigma) overnight, and subsequently cryoprotected using 25% sucrose in PBS. 100 μm coronal sections were cut using a sledge microtome and imaged under a Leica TCS SP5 confocal microscope for VSFP expression pattern.

For GEVI expression in fresh tissue slices, animals were terminally anaesthetised with ketamine/xylazine and transcardially perfused with ACSF^14^. 400 μm coronal sections were cut using a Leica vibratome and anatomical expression was imaged under an epifluorescence macroscope with a 1.0X objective.

### Voltage signal separation

All analysis was performed using custom-written analysis tools in MATLAB (2016b).

The voltage imaging signal was calculated as previously described^12,14^. In short, equalisation of heartbeat-related fluorescence modulation was first performed on both GEVI FRET acceptor (mKate2) and donor (mCitrine) signals, which effectively discounts vascular signal components such as heartbeat oscillations and haemodynamic-related slow fluctuations (<1 Hz) in both anaesthetised^14,27^ and awake animals^12,27^. Voltage signals were then calculated as the acceptor to donor ratio, resulting in ratiometric voltage map sequences. ΔR/R refers to (R-R_baseline_)/R_baseline_. R_basline_ is the value of R averaged over 10 images (~66 ms) directly preceding the stimulus. Photobleaching was negligible for the illumination intensity used (0.035 mW/mm^2^), thus no photobleaching correction was needed^14^.

### Generation of voltage map movies

The two cortical hemispheres were first registered to the surface of the Allen Institute Mouse Brain Atlas (www.brain-map.org) projected to our plane of imaging (Fig. 3, Supplementary Fig. S1B) using the centre of mass of initial responsive areas to visual (10 ms flashes of unstructured blue LED light) and whisker stimuli (as described above) as reference points^13,63^ registered into the corresponding centroids of VISp and SSp-bfd from ABM. ABM alignment with visual/somatosensory stimuli was calculated once for each mouse, yielding a reference alignment and an associated transformation matrix (non-rigid image alignment). Datasets from other imaging sessions of the same mouse were first registered to this reference alignment (rigid image alignment) based on its vasculature and then subjected to the corresponding transformation matrix. The functionally-registered membrane voltage maps were then averaged both across animals, datasets, and stimulus conditions (ie voltage map sequences from right-stimulation trials were left-right flipped) in the wakening or awake conditions, resulting in grand average ratiometric fluorescence movies (Movies S1 and S3). Spatial averages of the voltage map sequences across each cortical area were used to generate ROI movies cortical area maps colour-coded for population voltage activity (Figs 3 and 4, Supplementary Fig. S4; Movies S2 and S4). Frame times reported (Figs 3 and 4, Supplementary Fig. S4) are representative of the centre time of exposure.

### Quantification of response components

After functional registration to the Allen Brain Map (ABM), sensory-evoked responses for each cortical region were calculated by averaging across pixels allocated to those ROIs. Responses were first averaged across trials of each dataset. Latency (delay between stimulation and onset of initial depolarising response) was calculated from the time of intercept of the rising phase of the response and baseline. Amplitude of the of initial depolarising response was measured as the local maximum relative to baseline. The amplitude of the hyperpolarising response for the wakening condition was measured as the local minimum relative to baseline. The grand average time of these minima was used to calculate the amplitude of the repolarising response component for the awake condition.

### Statistical analysis

The computed measures for each measurement and dataset were used for statistical tests. For the awake and wakening conditions, mice were not imaged in both conditions within the same imaging session, thus we treated the datasets as independent measures and used unpaired Wilcoxon Mann-Whitney non-parametric comparisons that does not make assumptions on the distribution of data. Spearman’s rank correlation (two-tailed) was calculated for each region of interest for the trend in components of sensory evoked response. Binning over trials was employed as indicated.

### Data availability

The datasets analysed during the current study are available from the corresponding author on reasonable request.

## Electronic supplementary material

Movie 1

Movie 2

Movie 3

Movie 4

Supplementary Information
