# Supplementary material for: Cortical signatures of wakeful somatosensory processing
Source: Sci Rep. 2018 Aug 10;8:11977. doi: 10.1038/s41598-018-30422-9 (PMC6086870; doi:10.1038/s41598-018-30422-9)
Supplement: Supplementary file 5 — Supplementary Information [file 41598_2018_30422_MOESM5_ESM.pdf]

Cortical signatures of wakeful somatosensory processing

Chenchen Song, Denise M. Piscopo, Cristopher M. Niell, Thomas Knöpfel

SUPPLEMENTARY INFORMATION

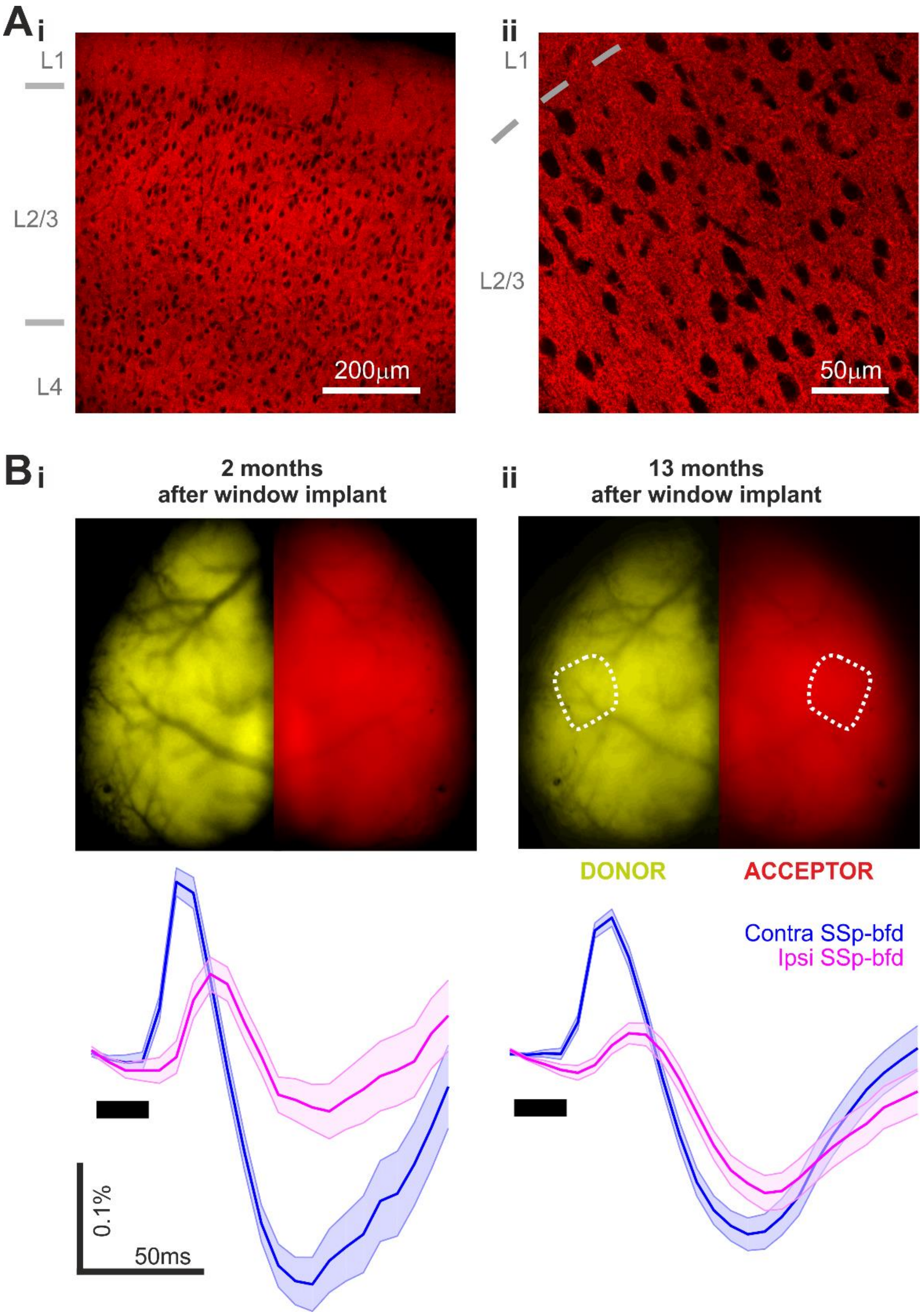

Supplementary Fig. S1. Transgenic GEVI imaging in vivo.

(A) Confocal images of coronal sections from CamK2A-tTA;TetO-chiVSFP mice after 4% paraformaldehyde fixation. chiVSFP is densely expressed across cortical layers (**i**), and indicator expression is targeted to the neuronal membranes (**ii**).
(B) Chronic transcranial dual-hemisphere preparation from one animal at 2 (**i**) and 13 (**ii** months following window implant. (**upper;** Note only the right hemisphere is shown and the donor image has been horizontally flipped for comparison at the two emission wavelengths). Dotted line outlines the primary somatosensory barrel cortex (SSp-bfd) after the functional registration to Allen Mouse Brain Atlas. Sensory-evoked responses from SSp-bfd to multi-whisker air puff shows functional stability over more than a year ΔR/R mean ± SEM).

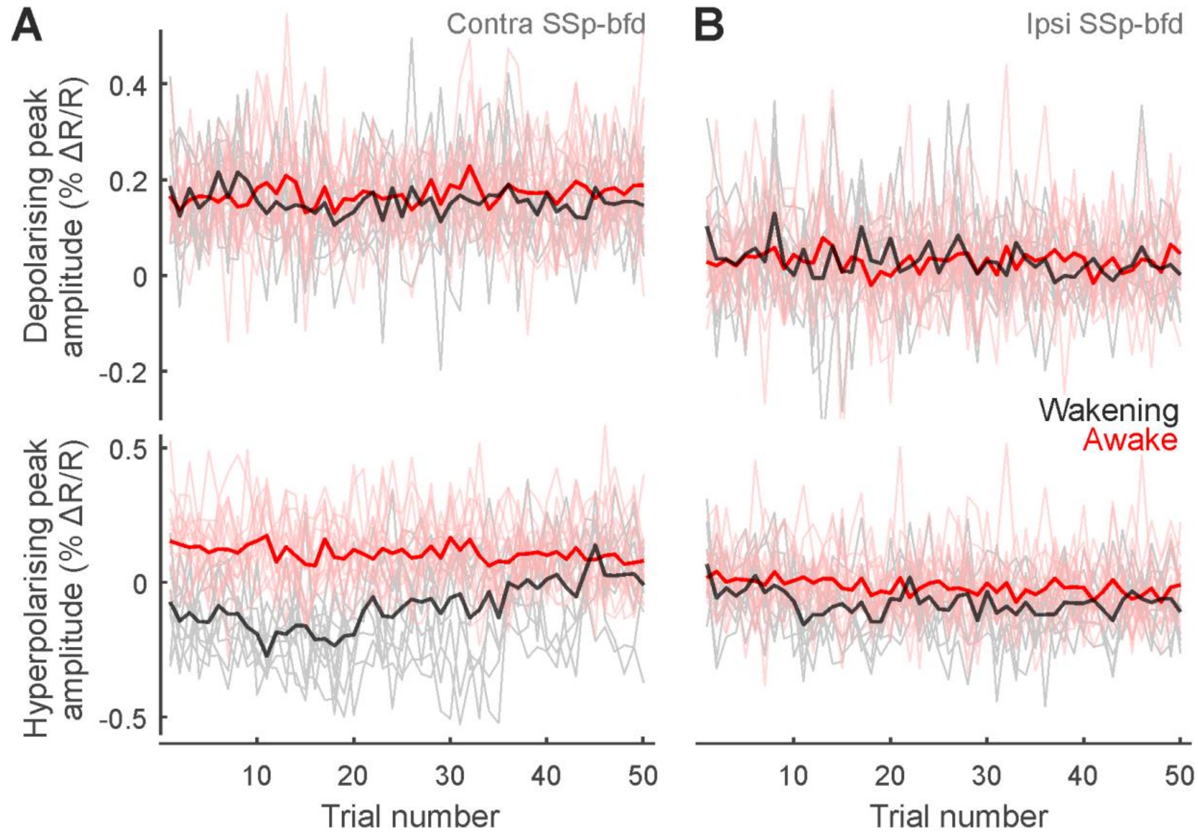

Supplementary Fig. S2. Altered response decay of sensory-evoked S1 pyramidal population across brain states due to hyperpolarising component.

(A) Depolarising amplitude of the initial peak response in contra SSp-bfd is constant both through the transition from wakening towards wakefulness (**upper**, black traces, Spearman's rank correlation (r_s) = -0.218, p = 0.128) and in the fully awake condition (**upper**, red traces, r_s = 0.060, p = 0.680). Faint traces: trace for individual 50-trial imaging datasets, solid traces: grand average.

For the wakening condition (N = 13 datasets, 5 mice), a main contributor to altered response decay component across brain states is the level of hyperpolarising activity at ~50 ms after the initial response (**lower**, black traces, r_s = 0.815, p < 0.001; *Note positive r_s value indicates reduction in hyperpolarising amplitude*) that decreases through waking up.

For the awake condition (N = 16 datasets, 5 mice), while depolarising amplitude remains comparable, the repolarising component increases (i.e. decrease in the measured amplitude; **lower**, red traces, r_s =-0.516, p < 0.001) indicating a potential decrease in alertness through the course of the awake imaging session.

(B) Similar comparisons for ipsi SSp-bfd.

For the wakening condition (N = 13 datasets, 5 mice), initial depolarising amplitude decreases modestly through the transition from wakening towards wakefulness (**upper**, black traces; r_s = -0.633, p < 0.001), with modest changes in hyperpolarising component (**lower**, black traces; r_s = 0.368, p = 0.009).

For the awake condition (N = 16 datasets, 5 mice), changes in initial depolarising amplitude is modest (**upper**, red traces; r_s = -0.338, p = 0.016), and an increase in repolarising/hyperpolarising activity is observed (**lower**, red traces; r_s = -0.697, p < 0.001).

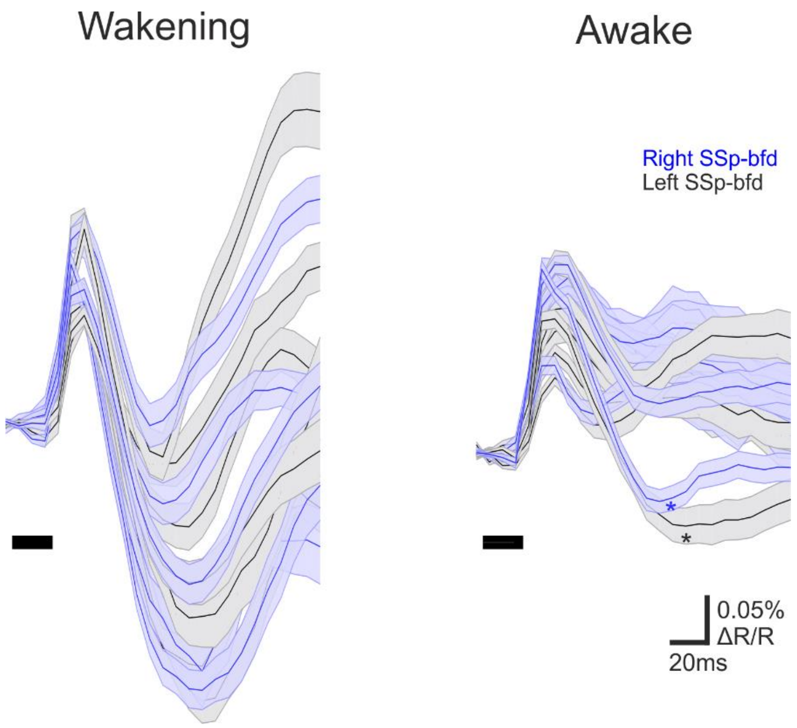

Supplementary Fig. S3. Whisker stimulation-evoked responses in left and right SSp-bfd.

Superimposed voltage imaging traces from left and right SSp-bfd in response to contralateral multi-whisker stimulation displays no lateralisation. (Wakening condition: N = 13 datasets, 5 mice; Awake condition: N = 16 datasets, 5 mice; mean ± SEM). Datasets for right-side stimulation (i.e. left SSp-bfd being the contralateral side) are left-right flipped after functional registration, and pooled for producing grand average. Note selected datasets in the awake condition that is reminiscent of the wakening condition (marked with asterisks, mouse may have fallen asleep); not excluded from analysis.

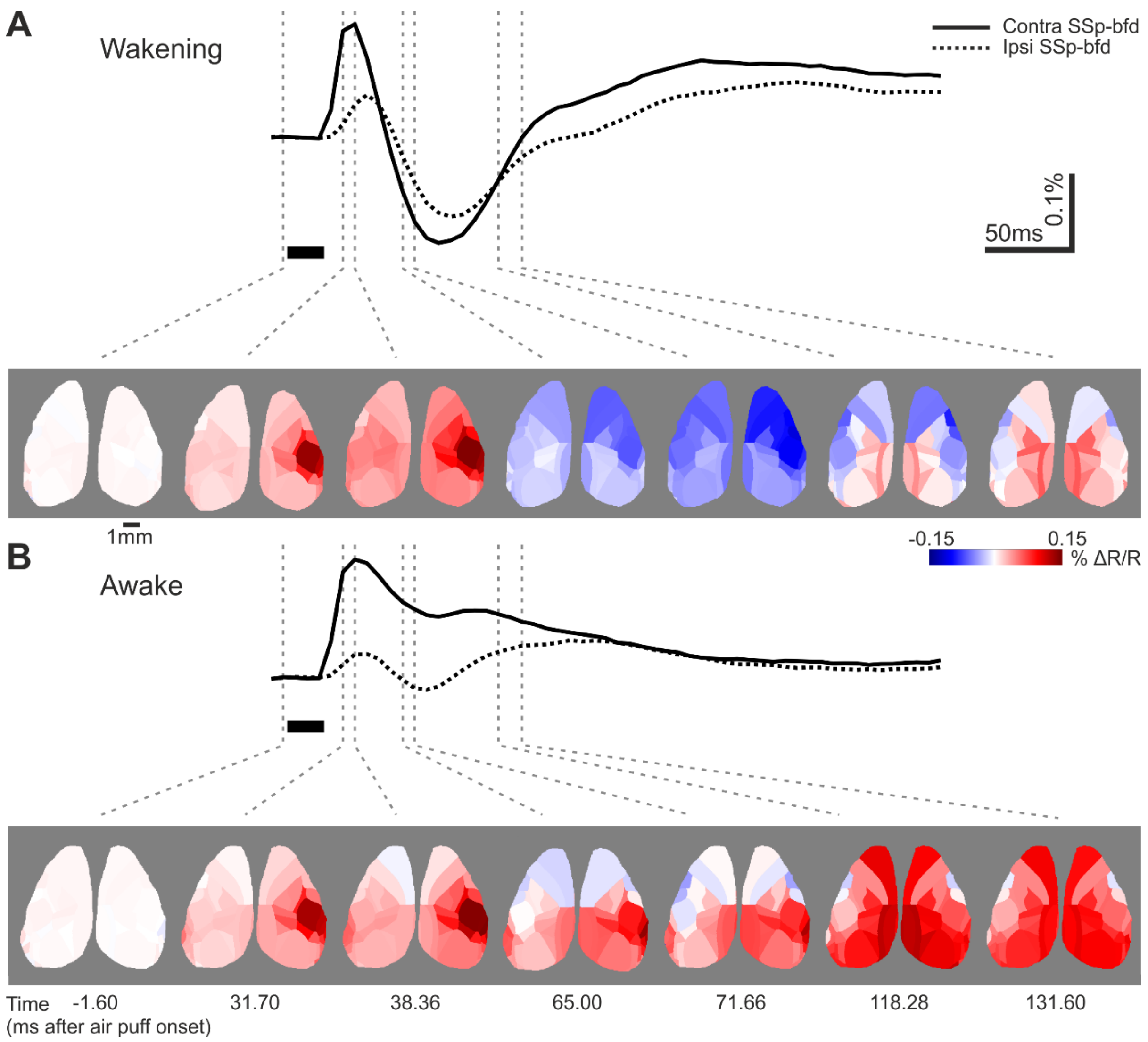

Supplementary Fig. S4. Selected response frames.

(A) Grand average trace from SSp-bfd (solid trace: contra; dashed trace: ipsi; Only average is shown for clarity) in the wakening state, and ratio movie frame with indication of selected time points as shown in Figure 3 (N = 13 datasets, 5 mice).
(B) Same as **(A)** for awake state (N = 16 datasets, 5 mice).

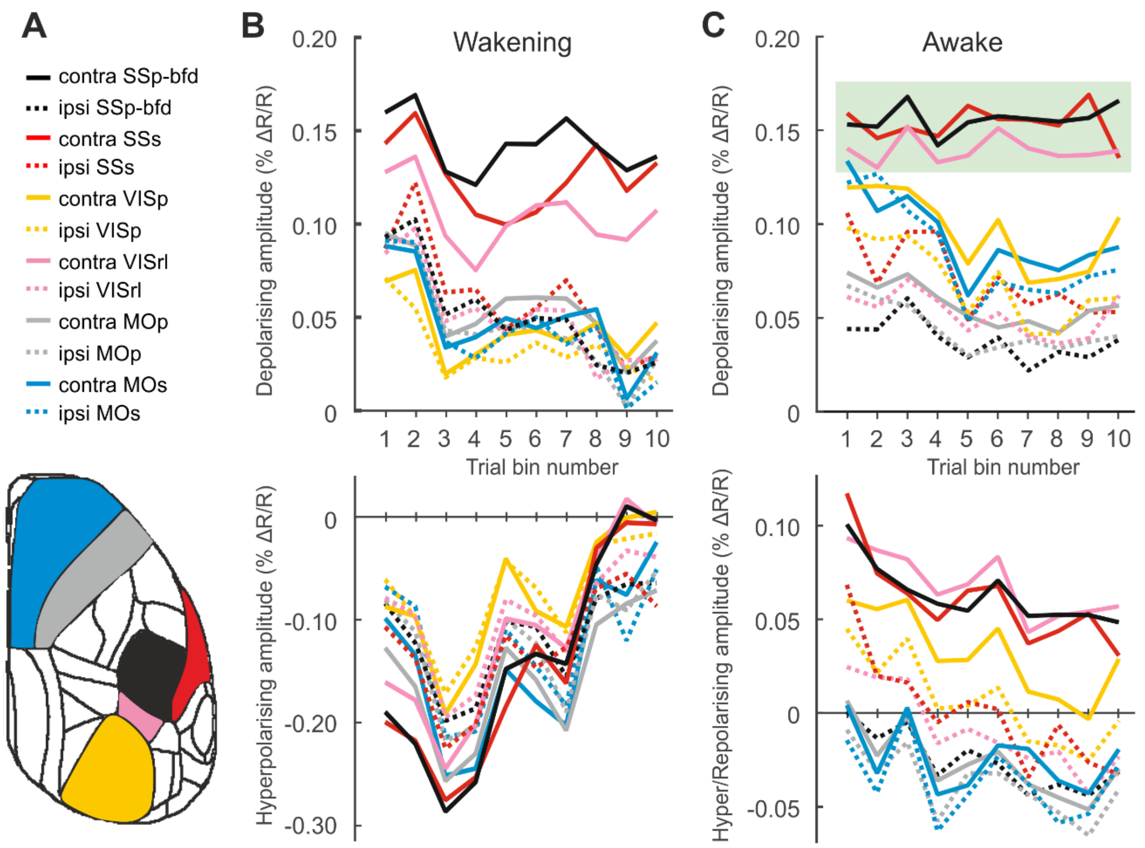

D

Key: positive r_s; negative r_s; $-0.1 < r_s < 0.1$; $p < 0.05$ (boxed)

	DEPOLARISING AMPLITUDE				HYPER/REPOLARISING AMPLITUDE			
	Wakening		Awake		Wakening		Awake	
	r_s	*Sig.*	r_s	*Sig.*	r_s	*Sig.*	r_s	*Sig.*
**contra SSp-bfd**	-0.218	*0.128*	0.060	*0.680*	0.815	*<0.001*	-0.516	*<0.001*
**ipsi SSp-bfd**	-0.633	*<0.001*	-0.338	*0.016*	0.368	*0.009*	-0.697	*<0.001*
**contra SSs**	-0.150	*0.299*	-0.072	*0.621*	0.789	*<0.001*	-0.457	*0.001*
**ipsi SSs**	-0.585	*<0.001*	-0.450	*0.001*	0.467	*0.001*	-0.582	*<0.001*
**contra VISp**	-0.183	*0.203*	-0.368	*0.009*	0.527	*<0.001*	-0.736	*<0.001*
**ipsi VISp**	-0.333	*0.018*	-0.438	*0.001*	0.442	*0.001*	-0.738	*<0.001*
**contra VISrl**	-0.198	*0.168*	-0.022	*0.878*	0.771	*<0.001*	-0.710	*<0.001*
**ipsi VISrl**	-0.606	*<0.001*	-0.264	*0.064*	0.417	*0.003*	-0.664	*<0.001*
**contra Mop**	-0.424	*0.002*	-0.380	*0.006*	0.477	*<0.001*	-0.597	*<0.001*
**ipsi MOp**	-0.481	*<0.001*	-0.390	*0.005*	0.301	*0.034*	-0.677	*<0.001*
**contra MOs**	-0.386	*0.006*	-0.348	*0.013*	0.417	*0.003*	-0.682	*<0.001*
**ipsi MOs**	-0.499	*<0.001*	-0.420	*0.002*	0.197	*0.171*	-0.687	*<0.001*

Supplementary Fig. S5. State-dependent response.

(A) Left: Selected ROIs in sensory and motor cortices on the Allen Brain Map, colour-coding used for traces shown. (abbreviations: SSp-bfd, Primary somatosensory area- barrel field; SSs, Supplemental somatosensory area; VISp, Primary visual area; VISrl, Rostrolateral visual area; MOp, Primary motor area; MOs, Secondary motor area)

(B) Upper - Grand average changes in amplitude of the initial depolarising response from selected ROIs (local maxima) from Left in the wakening condition (N = 13 datasets, 5 mice). 50 trials for each dataset is grouped into 10 bins of 5 trials per bin from each dataset, pooled from N to generate the grand average. Solid and dotted lines for contralateral and ipsilateral hemispheres respectively. (For clarity, only average traces are shown).

Lower – Same as Upper, for the hyperpolarising response component (local minima).

(C) Same as B for awake condition (N = 16 datasets, 5 mice). Green shading highlights the largely constant depolarising amplitude from contralateral SSp-bfd, SSs and VISrl, whilst depolarising response amplitude in other ROIs decreases through the imaging session, correlating with decreasing alertness. (For clarity, only average traces are shown).
Lower – Same as Upper, for the repolarising/hyperpolarising response component.
(D) Spearman's rank correlation of grand average trend changes in components of sensory-evoked response depicted in (**B**) and (**C**) for the selected ROIs in (**A**) through the dataset, for both wakening and awake conditions. Orange shading: positive r_s; blue shading: negative r_s; green shading: r_s between ±0.1; bold box outline: $p < 0.05$. *Note for hyperpolarising/repolarising amplitudes, positive r_s depicts a decreasing trend in this response component through the datasets.*

Supplemental Movies

Movie S1. Grand average ratiometric fluorescence movie of sensory-evoked pyramidal response under wakening condition (N = 13 datasets, 5 mice).

Movie S2. Grand average registered ROI regional average movie of sensory-evoked pyramidal response under wakening condition (N = 13 datasets, 5 mice).

Movie S3. Grand average ratiometric fluorescence movie of sensory-evoked pyramidal response under awake condition (N = 16 datasets, 5 mice).

Movie S4. Grand average registered ROI regional average movie of sensory-evoked pyramidal response under awake condition (N = 16 datasets, 5 mice).
